# Evaluation of the hepatotoxicity of the novel GPR40 (FFAR1) agonist CPL207280 in the rat and monkey

**DOI:** 10.1371/journal.pone.0257477

**Published:** 2021-09-23

**Authors:** Katarzyna Bazydlo-Guzenda, Pawel Buda, Mateusz Mach, Jerzy Pieczykolan, Izabela Kozlowska, Michal Janiszewski, Ewa Drzazga, Jakub Dominowski, Hubert Ziolkowski, Maciej Wieczorek, Shayne Cox Gad

**Affiliations:** 1 Innovative Drugs R&D Department, Celon Pharma S.A., Lomianki, Poland; 2 Postgraduate School of Molecular Medicine, Warsaw, Poland; 3 Department of Pharmacology and Toxicology, Faculty of Veterinary Medicine, University of Warmia and Mazury, Olsztyn, Poland; 4 Gad Consulting Services, Raleigh, North Carolina Area, United States of America; Zagazig University, EGYPT

## Abstract

GPR40 (FFAR1) is a promising target for the managing type 2 diabetes (T2D). The most advanced GPR40 agonist TAK-875 exhibited satisfactory glucose-lowering effects in phase II and III studies. However, the phase III studies of TAK-875 revealed drug-induced liver injury (DILI). It is unknown whether DILI is a consequence of a specific GPR40 agonist or is an inherent feature of all GPR40 agonists. CPL207280 is a novel GPR40 agonist that improves diabetes in Zucker Diabetic Fatty (ZDF) rats, Goto Kakizaki (GK) rats and db/db mice. In this report, the DILI-related toxicity of CPL207280 was compared directly with that of TAK-875. *In vitro* studies evaluating hepatic biliary transporter inhibition, mitochondrial function, and metabolic profiling were performed in hepatocytes from different species. The long term toxicity of CPL207280 was studied *in vivo* in rats and monkeys. Activity of CPL207280 was one order of magnitude lesser than that of TAK-875 for the inhibition of bile acid transporters. CPL207280 had a negligible effect on the hepatic mitochondria. In contrast to TAK-875, which was metabolized through toxic glucuronidation, CPL207280 was metabolized mainly through oxidation. No deleterious hepatic effects were observed in chronically treated healthy and diabetic animals. The study presents promising data on the feasibility of creating a liver-safe GPR40 agonist. Additionally, it can be concluded that DILI is not a hallmark of GPR40 agonists; it is linked to the intrinsic properties of an individual agonist.

## Introduction

GPR40 (FFAR1) has drawn attention as a potential target for T2D management. It is a G protein-coupled receptor (GPCR) for long and medium chain free fatty acids (FFAs) that is expressed in the membranes of β-cells in the islets of Langerhans. Activation of GPR40 amplifies glucose-dependent insulin secretion mainly via the Gα_q_-mediated pathway, which ensures regulation of glucose within the range of physiologically accepted concentrations [1]. Therefore, it is plausible that GPR40 agonists might supersede the popularity of currently administered sulfonylureas, owing to glucose dependence of the effect, minimizing the risk of hypoglycemic episodes. This promising GPR40’s *modus operandi* initiated pursue for synthetic, potent and selective compounds. Indeed, over the past two decades there have been many lead-molecules proposed as potential drugs including GW9508, TAK-875, AMG-837, AM-1638, AM-5262, TUG-424, TUG-770, LY2881835, JTT-851, P11187, SHR0534, AS2034178 [2–11]. In preclinical development, all of them displayed considerably greater potencies than FFAs and ensured pivotal glucose-dependent effects. The fraction of them accessed clinical trials but only few reached advanced phases [12, 13]. The latter are mainly represented by TAK-875 (Takeda), that proved efficacious in T2D patients and improved postprandial glucose and HbA1c levels, with a negligible risk of hypoglycemia, compared with those on glimepiride [14–16]. Further, positive outcomes were obtained for a similar compound MK-8666 (Merck) in clinical trials [17]. Despite successful glycemic control, both molecules were withdrawn from development due to indications of drug-induced liver injury (DILI). In phase I, TAK-875 was well tolerated following a single administration of increasing doses [18]. It was also well tolerated in a short (2 or 12 weeks) phase II study [16, 19]. Nevertheless, safety reports from numerous clinical sites during phase III studies were a cause for concern [20]. Serum alanine aminotransferase (ALT) elevations > 3 × upper limit of normal (ULN) cases rapidly increased in 2.7% of the study participants within six months; however, the same was not observed in the control group. This proportion was perceived as idiosyncratic DILI (iDILI) and therefore, the development was terminated. DILI is characterized by complex pathophysiology and has been a major reason for attrition in drug development [21]. To date, many drug properties and cellular mechanisms underlying DILI have been described [22, 23]. These include compound lipophilicity, production of reactive acyl glucuronide metabolite, oxidative stress, decrease in respiratory capacity of hepatic mitochondria, and inhibition of bile/xenobiotic transporters and cytochromes in the liver. TAK-875 has been studied in numerous *in vitro* and *in vivo* studies to identify its mechanism of toxicity. TAK-875 led to the impairment of bile acid transport due to inhibition of biliary transporters, such as bile salt export pump (BSEP), taurocholate co-transporting polypeptide (NTCP), organic anion-transporting polypeptides (OATPs), and multidrug resistance-associated proteins (MRPs). These malfunctions resulted in the accumulation of bile acids in hepatocytes and serum and accumulation of TAK-875 in the liver [24, 25]. TAK-875 induced mitochondrial dysfunction in hepatocytes by interfering with oxidative phosphorylation and reduction of glutathione content [24]. Similarly, TAK-875’s primary metabolite acyl-glucuronide was shown to enhance the toxicity of its parent drug, although, to a lesser extent. In a 14-day toxicology study in rats, TAK-875 showed a dose-dependent effect on ALT, total bile acids (TBA), and bilirubin (T-BIL) levels in serum. TAK-875 significantly increased ALT at doses of 200 and 600 mg/kg after seven days. Further, TAK-875 increased TBA and T-BIL levels at a dose of 600 mg/kg. The effect was less pronounced in dogs and was observed in only 2 of 6 animals treated with 600 mg/kg for 14 days. Collectively, all these pre and clinical studies have questioned whether the toxicity of GPR40 agonists is associated with a specific compound or is represented by the entire class represented by TAK-875.

CPL207280 is a novel GPR40 agonist that can stimulate the cognate receptor better than TAK-875. CPL207280 was carefully modeled and designed to minimize lipophilicity and molecular weight to address all potential safety concerns related to molecular properties [26]. To evaluate whether CPL207280 differs from GPR40 agonists, the hepatocytes toxicity due to the agonist treatment was compared with that of TAK-875 in *in vitro* assays. These included experiments, such as evaluation of human hepatocyte viability in 2D cultures, mitochondrial function and respiration in 2D and 3D cultures, function of human bile acid transporters, and metabolic profiling in hepatocytes of various species with a focus on potentially toxic reactive acyl-glucuronide. Next, toxicity was studied in a repeat-dose study in appropriate animal species, including rats and monkeys. These species were chosen based on similarity to human in both CPL207280’s metabolic elimination rate and metabolic profile when incubated with microsomes and hepatocytes. After ensuring similar pharmacokinetic (PK) dose-exposure relationships for CPL207280 and TAK-875, it was possible to compare pharmacodynamics, that is, liver safety markers and TBA and T-BIL levels in serum for both drugs in a 14-day study in rats [25].

## Methods and materials

### Materials

CPL207280 was designed and manufactured by Celon Pharma S.A., Poland. TAK-875 was synthesized by Celon Pharma Ltd., based on the reference compound TAK-875 by Takeda Ltd., Japan. Cryopreserved hepatocytes and microsomes used in the stability study were procured from Cyprotex Ltd. (Cheshire, UK).

### Viability assays

To study the viability of the liver cells, 5 × 10^3^ HepG2 (ATCC^®^) or human primary cells (ThermoFisher Scientific, USA) were seeded in 100 μL of DMEM media (Gibco; Thermo Fisher Scientific, USA) supplemented with fetal bovine serum (FBS)- in a 96-well plate. The following day, media was replaced with fetal bovine serum (FBS-)-free medium, and the following concentrations of the studied compounds were added in duplicates: 0.39, 0.78, 1.56, 3.125, 6.25, 12.5, 25, 50, and 100 μM. After 48 h, MTT test (Promega, USA) for HepG2 cells or RT-glo (Promega) tests for human primary cells were performed according to the manufacturer’s protocol. The experiments were performed in triplicates.

### Study of respiration in hepatocytes (Seahorse)

HepG2 cells (25 × 10^3^) were seeded in DMEM in special 8-well SeahorseXF plates coated with collagen (rat tail type 1 collagen: stock solution 3 mg/mL; Gibco, 125 μg/mL in 20 mM acetic acid; 50 μL/well (transparent bottoms). All procedures were performed in accordance with the Mito stress test user guide (Agilent Technologies, USA). Control compound concentration was as follows: Oligomycin, 2 μM; FCCP, 0.5 μM; Rotenone/Antimycin A, 0.5 μM.

### Stability in microsomes and cryopreserved hepatocytes

Williams E media supplemented with 2 mM L-glutamine, 25 mM HEPES, and the test compound (final substrate concentration 3 μM; final DMSO concentration 0.25%) were pre-incubated at 37°C, prior to adding a suspension of cryopreserved hepatocytes (final cell density 0.5 × 106 viable cells/mL in Williams E media supplemented with 2 mM L-glutamine and 25 mM HEPES) to initiate the reaction. The final incubation volume was 500 μL. Two control compounds (verapamil and donepezil) were included for each microsomal species, along with the appropriate vehicle control. The reactions were terminated by transferring 50 μL of incubate to 100 μL methanol containing an internal standard (metoprolol) at appropriate time points (0, 10, 20, 40, 60, and 120 min for the test compound and 0, 5, 10, 20, 40, and 60 min for the control compounds). The termination plates were centrifuged at 4000 x g at 4°C for 30 min to precipitate the protein. The supernatants were then collected for analysis, and LC-MS/MS was used for detection purposes. The system consisted of an Acquity^™^ Binary Solvent Manager, Acquity^™^ 4-position heated column manager, 2777 Ultra High Pressure Autosampler, and a Xevo-TQ MS triple quadrupole mass spectrometer (Waters Ltd., Herts, UK). This process was carried out twice.

### Metabolite profiling

Suspensions of cryopreserved human, rat, and monkey hepatocytes were pooled from a minimum of three individuals. All cryopreserved hepatocytes were purchased from a commercial supplier. Test or control compound at a concentration of 3 μM was incubated at 37°C. The cell density was 0.5 ×10^6^ viable cells/mL. The final DMSO concentration during the incubation was 0.25%. Control incubations were performed in lysed cells to reveal any non-enzymatic degradation. Two control compounds were identified in each species. Samples (50 μL) were removed from the incubation mixture at 0, 10, 20, 40, 60, and 120 min and added to methanol containing an internal standard (100 μL) to stop the reaction. The samples were centrifuged (4000 x g at 4°C for 30 min) and the supernatants at each time point were pooled for analysis by LC MS/MS using Cyprotex generic methods. Following hepatocyte stability assays, the samples were utilized for metabolite profiling. Metabolites were separated using a UPLC column (2.1 × 100 mm ACQUITY UPLC^®^ HSS T3 1.8 μm.). Metabolites were characterized using a mass spectrometer comprising Waters Xevo QT of G2-S, Acquity Binary Solvent Manager, Acquity Column Manager, and 2777 Autosampler.

### Transporters assay

In the vesicular transport inhibition assay (for BSEP, MRP) compounds were incubated with membrane vesicle preparations and the probe substrate. Incubations were carried out in the presence of 4 mM ATP or AMP to distinguish between transporter-mediated uptake and passive diffusion into the vesicles. For MRP2 and MRP3, reactions were carried out in the presence of 2 mM glutathione. Compounds were added to the reaction mixture in 0.75 μL of solvent (1% of the final incubation volume). The reaction mixtures were pre-incubated for 15 min at 37 ± 1°C. Reactions were initiated by the addition of 25 μL of 12 mM MgATP (or 12 mM AMP in assay buffer as a background control) and preincubated separately. Reactions were quenched by the addition of 200 μL of ice-cold washing buffer and immediate filtration via glass fiber filters mounted on a 96-well plate (filter plate). The filters were washed (5 × 200 μL of ice-cold washing buffer), dried, and the amount of substrate inside the filtered vesicles was determined by liquid scintillation counting.

For the uptake transporter inhibition assay, cells were washed twice with 100 μL of the appropriate buffer (HK, pH 7.4) for OATP1B1, followed by HBSS (pH 7.4) for (NTCP, OAT1, OAT2v1, OATP1A2, and OATP1B3). Uptake experiments were carried out at 37 ± 1°C in 50 μL of the respective buffer containing the probe substrate and the compound or solvent. The organic solvent concentration was equal in all wells and did not exceed 1.5% (v/v). After the experiment, cells were washed twice with 100 μL of cold appropriate buffer and lysed with 50 μL of 0.1 M NaOH. Radiolabeled probe substrate transport was determined by measuring an aliquot (35 μL) from each well for liquid scintillation counting.

### Three dimensional spheroids

Hepatic spheroids were formed by seeding HepaRG^™^ cells into round-bottom ultra-low adhesion 96-well spheroid plates (Corning^®^) in 3D liver media with 0.5% DMSO, 100 U/mL penicillin, and 100 mg/L streptomycin. Cells were incubated at 37°C and 5% CO_2_ until spheroid formation and then compound-treated. The test compound was diluted in the vehicle, and serial dilutions were made in a 0.5% vehicle in HepaRG^™^ induction media. Eight concentrations of the test compound in triplicate were then incubated for 14 days with re-dosing on days 4, 7, 10, and 13 (16 h prior to assay) of compound exposure. L-buthionine sulfoximine was used as positive control for GSH content, whereas rotenone was simultaneously used as a positive control for mitochondrial dysfunction and ATP content. At the end of the incubation period, the cells were loaded with the relevant dye for each cell health marker: DNA structure–Hoescht, GSH content–mBCL, ROS formation–DHE, mitochondrial dysfunction–MitoTracker, and cellular ATP content–CellTiter-Glo^®^, Promega. The plate was then scanned using an automated confocal fluorescent cellular imager, ArrayScan^®^ XTI (Thermo Scientific Cellomics) at 37°C and 5% CO_2_. Further, the cellular ATP content was measured using CellTiter-Glo^®^ (Promega, USA).

### Pharmacokinetics in rats and monkeys

Animals were cared for in accordance with the principles outlined in the current "Guide to the Care and Use of Experimental Animals" as published by the Canadian Council on Animal Care and the "Guide for the Care and Use of Laboratory Animals,” a NIH publication [27]. The study was approved by the ethics committee of the institution (ITR Laboratories, Canada).

Each rat and monkey was bled by venipuncture at specific times after administration and the samples were collected into tubes containing the anticoagulant K_2_EDTA. The tubes pending processing were placed on wet ice. Following collection, the samples were centrifuged (1200 × g for 10 min at approximately 4°C) within 60 min of collection, and the resulting plasma was recovered and split into two aliquots of approximately equal volume (minimum 100 μL of plasma in each aliquot). The analyte (CPL207280) and its internal standard (CPL207280-d7) were extracted from a 25.0 μL aliquot of the animal K_2_EDTA plasma using a semi-automated protein precipitation extraction. The extracted samples were injected into a high-performance liquid chromatography (HPLC) system (Shimadzu LC-20AD pumps and Shimadzu SIL-20AC autosampler) connected to a Hypersil Gold PFP, 50 × 4.6 mm, 5 μm column. The mobile phase consisted of 2 mM ammonium acetate with 0.05% acetic acid and methanol. The gradient chromatographic separation using a flow rate of 1.00 mL/min was at a nominal temperature of 28°C. The detection was carried out using an API 5000 triple quadrupole mass spectrometer (Applied Biosystems). The calibration range for this assay ranged from 10.0 to 2000.0 ng/mL.

### Pharmacokinetics in mice

All procedures on mice were approved by the local ethics committee (approval no.: 45/2017 from 27.07.2017 Experimental Center of the Medical University in Bialystok, Poland) and were conducted in accordance with EU Directive 2010/63/EU for animal experiments [28]. The study was performed in 128 male C57BL6/cmdb mice, 20–25 g, 8–10 weeks old. The mice were separated into four groups of 32 mice per group. The animals were fasted for 12 h (water *at libitum*). At time point t = 0, animals received a single dose of the compounds orally. Each compound was prepared in a formulation of 5% DMSO/55% PEG/40% citrate buffer pH 3 and administered at a dose of 3 mg/kg. Four hours after drug administration, the animals received standard chow. At each of the eight time points (15 min, 30 min, 1 h, 2 h, 4 h, 7 h, 12 h, and 24 h), four mice in each group were euthanized and the liver of the euthanized mice was collected. After solubilization in extraction buffer, the solutions were shaken for 1 min and then centrifuged for 4 min (4000 x g). Supernatants were collected and analyzed HPLC (Agilent Technologies 1260, Sciex QTrap 4500).

### Healthy animals in the toxicology study

#### Rats

Forty-seven male and forty-seven female Wistar Han rats were procured from Charles River Kingston (3121 US Highway 209, Stone Ridge, NY 12484, USA). Rats were cared for in accordance with the principles outlined in the current “Guide to the Care and Use of Experimental Animals” as published by the Canadian Council on Animal Care and the “Guide for the Care and Use of Laboratory Animals,” a NIH publication. The study was approved by the ethics committee of the institution (ITR Laboratories, Canada). At baseline, the rats were approximately 8 to 9 weeks old. The body weights of the rats ranged from 207 to 239 g for males and 164 to 188 g for females. The test and control/vehicle items were administered the test compounds for 14 or 56 consecutive days by oral gavage, using a gavage needle attached to a syringe. The dose volume was 10 mL/kg for all animals. The actual volume administered to each rat was calculated and adjusted based on the most recent practical body weight of each animal.

#### Monkeys

Eight male and eight female Cynomolgus monkeys (Macaca fascicularis), as well as one spare monkey/sex, were received from Worldwide Primates, Inc. (16450 S 180 ST, Miami, FL USA 33187). Monkeys were handled in accordance with the principles outlined in the current “Guide to the Care and Use of Experimental Animals” as published by the Canadian Council on Animal Care and the “Guide for the Care and Use of Laboratory Animals,” a NIH publication. The study was approved by the ethics committee of the institution (ITR Laboratories, Canada). At baseline, the monkeys were approximately 2 to 3 years old. The body weights of the monkeys ranged from 2.3 to 3.1 kg for males and 2.2 to 3.2 kg for females. Approximately 1 h prior to dosing, the animals were water and food-deprived. Water and food were resumed at 60 min post dosing. The test and control/vehicle were administered by oral gavage for 14 or 56 consecutive days, using a gavage tube attached to a syringe. The dose volume was 5 mL/kg for all animals. The gavage tube was rinsed with approximately 4 mL of reverse osmosis water to ensure that the entire dose was delivered. The actual volume administered to each monkey was calculated and adjusted based on the most recent practical body weight of each animal.

For the pharmaco- and toxico-kinetic study, animals (unanesthetized) were bled from the jugular venipuncture. Further, the samples were collected into tubes containing the anticoagulant K_2_EDTA. For gross examination and organ weight study, all animals were euthanized upon administration of the last dose, following an overnight period without food. These animals were anesthetized with isoflurane (rats) or ketamine (monkeys) to allow collection of blood samples for clinical pathology evaluation, followed by exsanguination. In monkeys, euthanasia via an intravenous overdose of sodium pentobarbital was followed by exsanguination resulting from the transection of major blood vessels. The necropsy consisted of external examination, including references to all clinically recorded lesions as well as a detailed internal examination.

### Diabetic animals

#### Rats

Male ZDF rats (n = 8) and ZL rats (n = 8) were aged 9 weeks and were housed 2–3 per cage within a small animal facility in accordance with the guidelines approved by the Association for Assessment and Accreditation of Laboratory Animal Care. The study was approved by the ethics committee of the institution (CrownBio, China). All animals had *ad libitum* access to water during the entire study period. The animals were fed a diet of Purina Rodent LabDiet 5C08, equivalent to LabDiet 5008. Treatment-grouped rats (N = 8) underwent oral gavage with the test compound or vehicle at 10 mL/kg for 6 weeks. For fasting blood levels of ALT and AST after overnight fasting, blood was collected from the jugular vein or through a cardiac puncture (on the day of euthanasia, the last day of the study). Blood was collected in AXYGEN microtubes (MCT-150-C) for clinical blood chemistry measurements. Next, the sample was allowed to clot for a minimum of 30 min at room temperature and then centrifuged at 4°C and 4000 x g for 10 min. The resultant serum was transferred into three pre-labeled AXYGEN microtubes for blood chemistry analysis using the ADVIA 2400 chemistry system. Rats were euthanized by overdose of CO_2_ followed by decapitation.

#### Monkeys

All animals aged 8–20 years had *ad libitum* access to water and were fed twice daily with a complete nutritionally balanced diet (Beijing Keao Xieli Feed Co., LTD, Beijing, China) enriched with seasonal fruits. The cynomolgus monkeys used in the study were cared for and handled in accordance with all applicable Association for Assessment and Accreditation of Laboratory Animal Care regulations and guidelines. The study was approved by the ethics committee of the institution (CrownBio, China). After each treatment (weighing, bleeding, or dosing), the animals were clinically observed on the day of the procedure and, if previously anesthetized, the animals were monitored to stand up and alert. Nine selected diabetic animals were subjected to daily test compound (powder) dosing via natural feeding with bananas (n = 3). Blood from overnight-fasted animals was collected weekly in the morning for measurement of serum ALT and AST levels on days 0, 7, 14, 21, 28, and 35. Blood samples (1.5 mL) were collected from a cephalic or saphenous vein into BD Vacutainer^®^ and K_2_-EDTA tubes.

## Results

### Viability of hepatocytes

**Fig 1:**
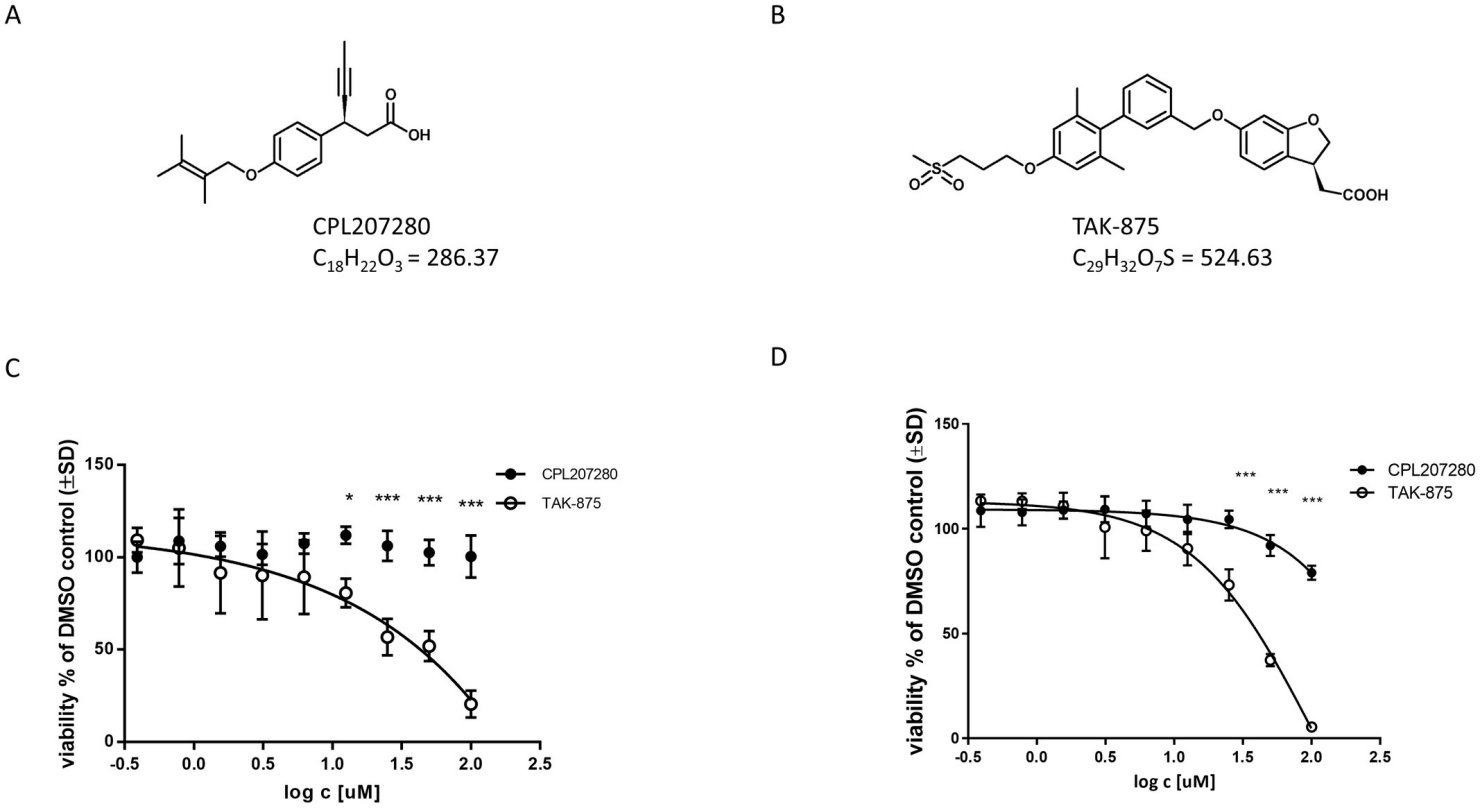
Comparison of CPL207280 and TAK-875 structures and hepatotoxicity *in vitro*. Structures of CPL207280 (A) and TAK-875 (B) and viability of HepG2 cells (C) and primary human hepatocytes (D) incubated with a range of concentrations of CPL207280 and TAK-875 for 48 hours. Values are normalized to appropriate DMSO-treated controls. Data denote mean ± SD. Statistical analysis was performed using Two-Way ANOVA followed by Sidak’s post-hoc test; n = 3; ***,p < 0.001; **, p < 0.01; *, p < 0.5.

CPL207280 in contrast to TAK-875 was designed to reduce molecular weight and lipophilicity whereby minimize the risk of liver toxicity. This was enabled by introduction of small acyclic motif bound to aromatic structure. Concomitantly, the chiral carbon residue between carboxyl and aromatic groups enhanced selectivity and potency (Fig 1A and 1B) [26, 29]. To study the potential toxicity in the liver, HepG2 and human primary hepatocytes were incubated with CPL207280 and TAK-875 for 48 h, and their viability was measured. Neither compound exhibited altered viability at concentrations up to 10 μM (Fig 1C and 1D). At 100 μM, TAK-875 abolished the growth of HepG2 and primary cells. In contrast, CPL207280 reduced viability by only 20%, suggesting a broader safety window for future studies.

### The effect of CPL207280 and TAK-875 on hepatic mitochondria

**Fig 2:**
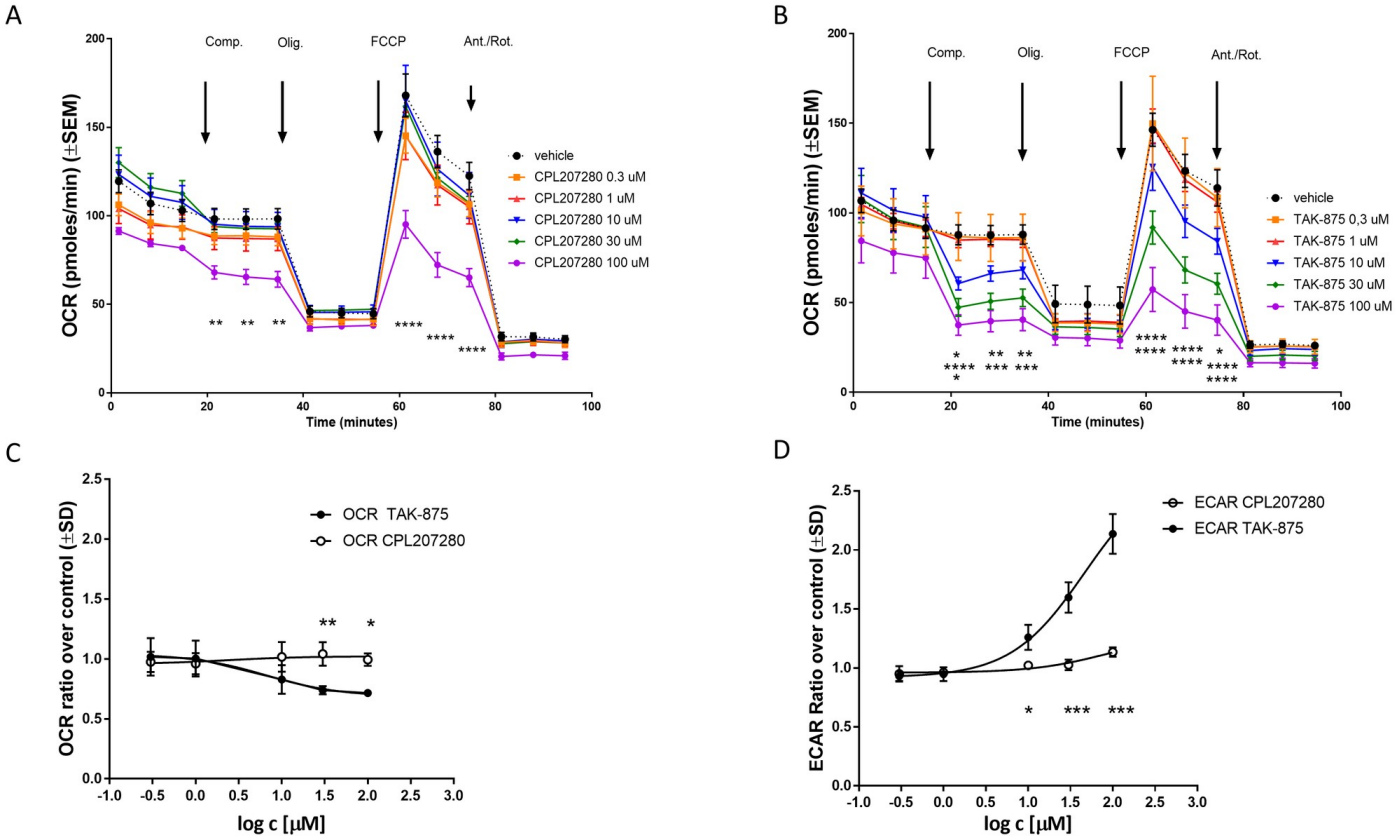
The effect of CPL207280 and TAK-875 on hepatocyte respiration. Representative tracing of oxygen consumption rates following a mitochondrial stress test in HepG2 cells treated with CPL207280 (A) and TAK-875 (B). Basal Oxygen Consumption rate (C) and Extracellular Acidification Rate (D). Statistical analysis was made by Two-Way ANOVA followed by Sidak’s post-hoc test. ***, p< 0.001; **, p< 0.01; *, p< 0.05; n = 3.

To study whether both compounds may interfere with oxidative phosphorylation, as reported for TAK-875, [24, 25] respiration was studied in HepG2 cells using the Seahorse XF platform. The oxygen consumption rate (OCR) decreased dramatically after injection of TAK-875 at a concentration of 10 μM and higher, thus indicating its impact on basal respiration (Fig 2B). Addition of oligomycin, an ATP synthase inhibitor, reduced ATP synthesis. Following the addition of FCCP, an uncoupler of mitochondrial oxidative phosphorylation, TAK-875 showed a dose-dependent reduction in maximal respiratory capacity. In contrast, CPL207280 reduced basal and maximal respiration at the highest concentration of 100 μM and did not alter ATP synthesis (Fig 2A and 2C). Moreover, TAK-875 induced acidification of media with EC_50_ = 46 μM, suggesting a switch from oxidative to non-oxidative respiration. This effect was absent for CPL207280 over the entire range of concentrations (Fig 2D). All these results were suggestive of mitochondrial damage caused more prominently by TAK-875.

### CPL207280 and TAK-875 act differently on mitochondria of hepatic 3D spheroids

Next, mitochondrial defects caused by TAK-875 and CPL207280 in a 3D spheroid system were comprehensively analyzed. Spheroids composed of HepaRG^™^ cells are structurally similar to real hepatic tissue in contrast to monolayer clonal or primary cells. They comprise three-dimensional (3D) cell cultures, which allow for better recapitulation of the complex in an *in vivo* microenvironment than traditional 2D monolayer models [30]. They also allow the treatment duration to be extended from hours to days. Therefore, they offer more valuable data than classic 2D cell cultures. Spheroids were exposed to increasing concentrations of test compounds (0.05–100 μM) for 14 days. Further, they were monitored for various cellular and mitochondrial health markers such as spheroid count, spheroid size, DNA structure, mitochondrial mass, mitochondrial membrane potential (Δψm), oxidative stress, glutathione content, and cellular ATP. Neither compound altered the spheroid count or spheroid size (Table 1). TAK-875 impaired DNA structure, decreased mitochondrial mass, dose-dependently decreased mitochondrial membrane potential, increased reactive oxygen species (ROS), and reduced cell glutathione content in a dose-dependent manner. In contrast, CPL207280 did not affect these markers within the same range of concentrations. TAK-875 potently reduced cellular ATP content, whereas only a slight impact was observed with administration of CPL207280. Thus, TAK-875 interferes with many vital cellular processes at relatively low concentrations, not greater than 10 μM. CPL207280 requires concentrations of at least one order of magnitude greater than TAK-875 to induce a significant toxic effect.

**Table 1:** Effects of CPL207280 and TAK-875 on pivotal hepatocyte health markers.

Parameter	Estimated AC_50_ (μM) (95% CI of AC_50_)	
	CPL207280	TAK-875
Spheroid count	NR	NR
Spheroid size	NR	NR
DNA structure	NR	34.2 (21.6 to 54.6)
Mitochondrial mass	NR	>100
Mitochondrial membrane potential	NR	56.4 (38.8 to 83.9)
Oxidative stress	NR	>100
Glutathione content	NR	54.7 (31.7 to 99.1)
Cellular ATP	>100	30.1 (19.7 to 46.6)

HepaRG^™^ spheroids were loaded with fluorescent dyes to detect the studied markers and then incubated with test compounds for 14 days at concentrations up to 100 μM. Data represent calculated AC_50_, that is, the concentration at which a 50% maximum effect is observed for each cell health parameter, unless the effect was negligibly low so that AC_50_ was impossible to assess—NR (not reduced).

### Comparison of stability of CPL207280 and TAK-875 in microsomes and hepatocytes of different species

GRP40 agonists are considered compounds that are quickly metabolized, which can be problematic for development of drugs with desired, sustainable effect. TAK-875 was designed to ensure a relatively long T_1/2_ and slow clearance rate in humans, which resulted in its long exposure (approximately 30 h) in humans [18]. To compare the rate of CPL207280 metabolism with that of TAK-875, the compounds were incubated with microsomes (phase I) and hepatocytes of different species: mouse, rat, dog, monkey, and human. The second aim of this experiment was to select animal species for further toxicity studies, that is, species, which ensure a metabolic rate of CPL207280 similar to that of humans. As expected, of all the tested species, metabolism rate of TAK-875 observed in dog and rat microsomes was similar to that of humans (Table 2). This was in line with previous reports and supports the choice of animal species for TAK-875 treatment in a previous toxicology study [31].

**Table 2:** Stability of CPL207280 and TAK-875 in the presence of microsomes from different species.

Compound	Rat (Sprague Dawley)		Mouse (CD)		Human		Dog (Beagle)		Monkey (Cynomolgus)		Mini pig	
	Cl_int_ [μl/min/mg]	T_1/2_ [min]	Cl_int_ [μl/min/mg]	T_1/2_ [min]	Cl_int_ [μl/min/mg]	T_1/2_ [min]	Cl_int_ [μl/min/mg]	T_1/2_ [min]	Cl_int_ [μl/min/mg]	T_1/2_ [min]	Cl_int_ [μl/min/mg]	T_1/2_ [min]
CPL207280	21.1	69	11.9	116	49.2	28	17.1	81	60.9	23	45	31
TAK-875	11.6	119	4	343	6.6	210	12.4	112	39.2	30	91.1	15
Intrinsic Clearance Cl_int_ [μl/min/mg protein] *												
Stable compound	< 13.2		< 8.8		< 8.6		<5.3		<12.5		< 13.2	
Unstable compound	>71.9		> 48		> 47		>28.9		>67.8		>71.9	

The test compounds were incubated with microsomes for 0, 10, 20, 40, 60, and 120 min, after which the supernatant was subjected to LC-MS/MS analysis for quantitative assessment of test compounds. Data represent the mean half-life of the parent compound, T_1/2_, and mean intrinsic clearance, CL_int_ (n = 2). Based on the mean clearance data, the extent of metabolism from greatest to least was monkey ≈ human >> dog ≈ rat > mouse.

* Reference ranges of values by Cyprotex.

Based on the values obtained from different species, TAK-875 was classified as a stable compound. CPL207280 proved to be an unstable compound and was metabolized at a high rate by human and monkey microsomes. Microsomes offer only limited insight into metabolism (usually phase I and II) because they possess only a fraction of enzymes. To obtain a comprehensive insight into the metabolic rate, the next study was performed in hepatocytes, equipped with all enzymes involved in liver metabolism. Again, CPL207280 showed high clearance rates for humans and was similar for monkeys (Table 3). These results contributed to the final decision on choosing monkeys, besides rats, as a second species in a repeat-dose toxicity study of CPL207280. Note that for TAK-875, rats were chosen as the first species, while the second species was the dog. Hence, the *in vivo* comparison of the toxicity of CPL207280 and TAK-875 was enabled only in rats.

**Table 3:** Stability of CPL207280 and TAK-875 in the presence of hepatocytes from different species.

Compound	Rat (Sprague Dawley)		Mouse (CD)		Human		Dog (Beagle)		Monkey (Cynomolgus)	
	Cl_int_ [μl/min/mg]	T_1/2_ [min]	Cl_int_ [μl/min/mg]	T_1/2_ [min]	Cl_int_ [μl/min/mg]	T_1/2_ [min]	Cl_int_ [μl/min/mg]	T_1/2_ [min]	Cl_int_ [μl/min/mg]	T_1/2_ [min]
CPL207280	7.51	186	3.46	404	82.8	17	7.76	180	81.4	17

Test compounds were incubated with hepatocytes for 0, 10, 20, 40, 60, and 120 min, after which the supernatant was subjected to LC-MS/MS analysis for quantitative assessment of test compounds. Data represent the mean half-life of the parent compound, T_1/2_, and mean intrinsic clearance, CL_int_ (n = 2). Based on the mean clearance data, the extent of metabolism from greatest to least was monkey ≈ human >> dog ≈ rat > mouse.

### Glucuronide metabolite of CPL207280 is produced in negligible amounts in the human and rat hepatocytes

**Fig 3:**
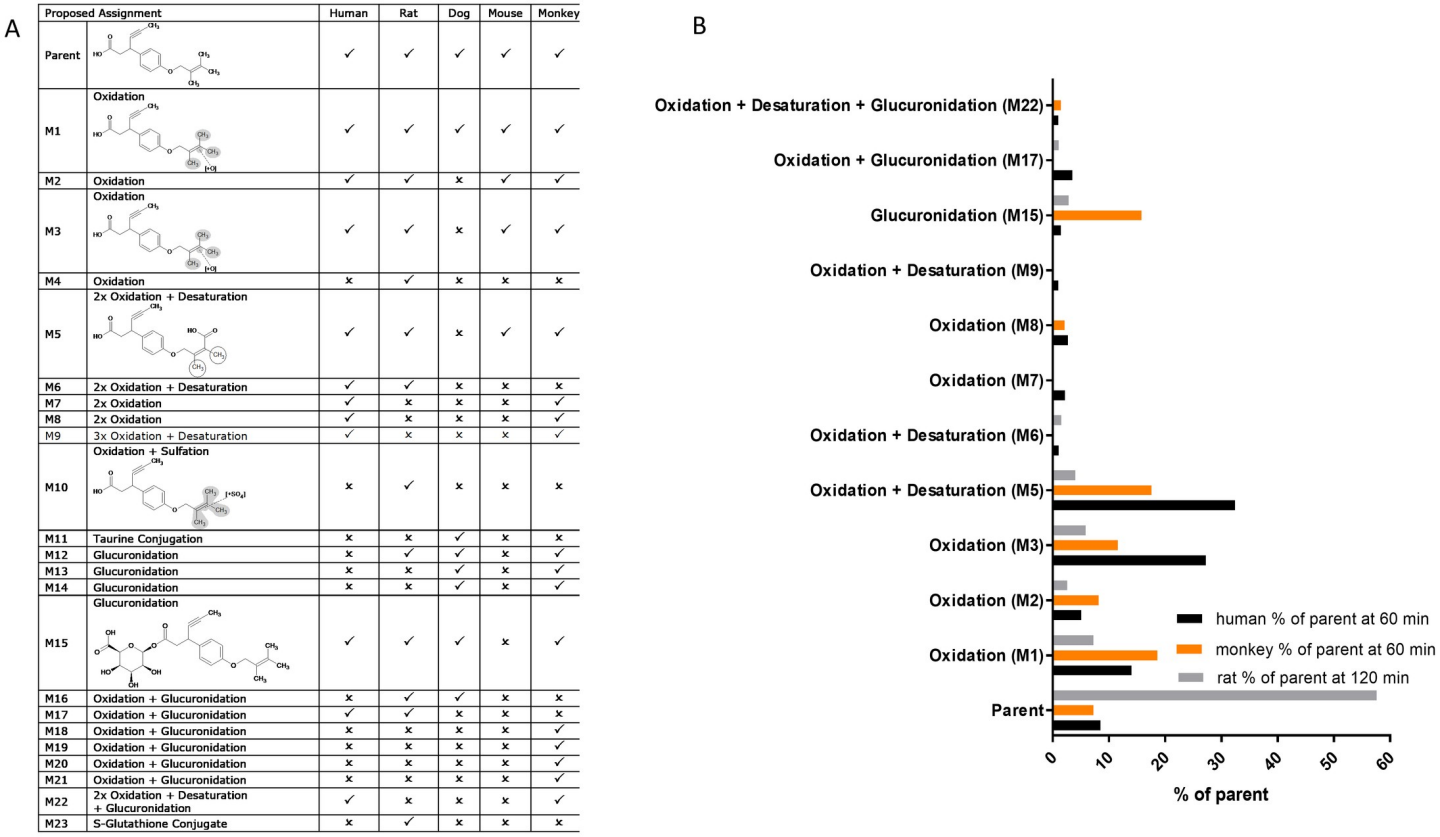
Metabolic profiling of CPL207280. Qualitative characterization of subsequent metabolites (A) and quantitative analysis (B). Data show the percent of the parent compound after incubation time. Human, monkey and rat hepatocytes were exposed to 10 μM concentration of the compound for the time determined by T_1/2_ in the given species. That is: for 60 minutes for human and monkey and 120 minutes for rat hepatocytes. Next, the supernatant was collected and metabolic changes of the parent compound were identified using UPLC followed by LC-MS/MS.

A number of carboxylic acid-containing drugs have been withdrawn from the market due to adverse effects stemming from the formation and reactivity of acyl glucuronide metabolites (for review [32]). It is hypothesized that reactive acyl glucuronides form protein adducts via both trans-acylation and glycation mechanisms. To analyze whether CPL207280 is bioprocessed to glucuronide metabolites, metabolite profiling was performed in primary hepatocytes from different species: humans, rats, mice, monkeys, and dogs. To that end, hepatocytes from different species were incubated with 10 μM CPL207280 for the time course justified by the previously established T_1/2_ of the compound for each species. Hence, the maximal incubation time was 120 min for rats, mice, and dogs, and 60 min for humans and monkeys. In all species, 23 metabolites were identified, of which 12 were produced by human hepatocytes (Fig 3A). Monkey hepatocytes produced nine, seven, four, and two human metabolites. The analysis revealed that CPL207280 undergoes oxidation, desaturation, reduction, glucuronidation, and glutathione binding (the latter only in rat hepatocytes). In human hepatocytes, the main products that emerged after 60 min of incubation resulted from oxidation and accounted for 85.7% of the amount of the parent compound (Fig 3B). Glucuronidation gave rise to three products comprising 6.0% of the parent amount (M15, M17, and M22). Human and rat hepatocytes produced negligible amounts of glucuronide. The largest amount of glucuronide (M15) was observed in monkeys (15.8% of the parent amount). Collectively, of all tested species, monkey and rat generated metabolites that covered all the metabolites found in humans and that there were no metabolites unique for human. These results, supported by the metabolic-rate study suggested that rats and monkeys are the suitable species for further toxicology studies. As TAK-875 was previously studied in rats and dogs, only rats turned out to be a common platform for comparison of both compounds *in vivo*.

### CPL207280 shows little propensity to cause bile acid transporters inhibition

**Fig 4:**
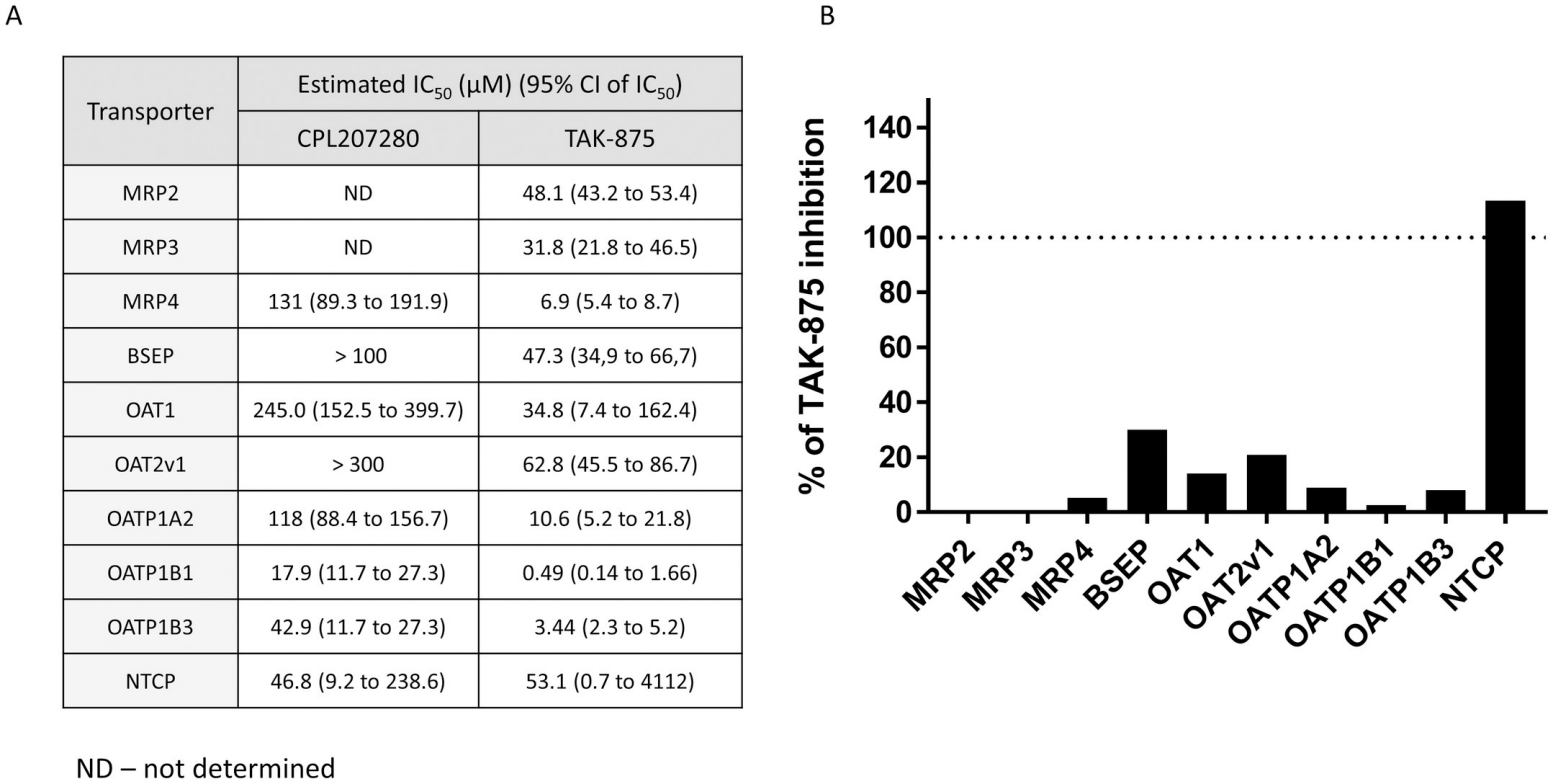
Inhibition of bile acid transporters by test compounds CPL207280 and TAK-875. The transformed HEK cells or vesicles expressing indicated transporters were incubated with a range of concentrations (up to 300μM) of test compounds for 1 hour. Next, the inhibition of substrate specific for each transporter was measured. Data represent mean IC_50_ (A) and percent of inhibition for CPL207280 relative to that of TAK-875 (B); n = 2.

Different types of bile acids (BAs) are produced by the liver from cholesterol and are transported to the bile and further to the upper intestines to facilitate digestion of foods. Next, they move along with the digested nutrients down the gut and are reabsorbed in the ileum, wherein they enter the blood and return to the liver. This process is called enterohepatic circulation and was found to be impaired in the livers of rats and dogs treated with TAK-875 [25]. The causative mechanism of this impairment was identified as TAK-875 and its glucuronide metabolite–driven inhibition of crucial BA transporters in the liver. These include liver uptake transporters Na^+^-taurocholate co-transporting polypeptide (NTCP), organic anion transporting polypeptides (OATPs), liver export transporters, bile salt export pump (BSEP), and multidrug resistance-associated proteins (MRPs). In particular, inhibition of the BSEP transporter has been well characterized in pathological states and pharmacology. Blockade of BSEP results in retention of bile acids, which culminates in cholestasis and contributes to DILI [33, 34]. There are, however, compensatory mechanisms provided by other transporter isoforms; therefore, susceptibility of the drug for DILI increases as the drug targets more isoforms [35]. We studied the BA transporters that are inhibited by TAK-875, and by CPL207280 *in vitro* (Fig 4A). As expected, TAK-875 inhibited BSEP at low concentrations. In contrast, CPL207280 inhibited BSEP at higher concentrations, resulting in greater IC_50_ values. CPL207280 showed no affinity to MRP2 and MRP3 transporters, whereas TAK-875 showed IC_50_ of 48.1 and 31.8 μM, respectively. CPL207280 demonstrated at least 10-fold less inhibitory potency than TAK-875, for MRP4 efflux transporters, and influx transporters including OAT1, OAT2v1, OATP1A2 and OATP1B1. The only similarity was observed for the NTCP influx transporter (Fig 4B). Inhibition of the NTCP influx transporter may lead to the blockade of recirculation of total bile acids (TBA) and bilirubin, and in consequence their elevated levels in the bloodstream.

### Comparison of pharmacokinetic parameters of CPL207280 and TAK-875

**Fig 5:**
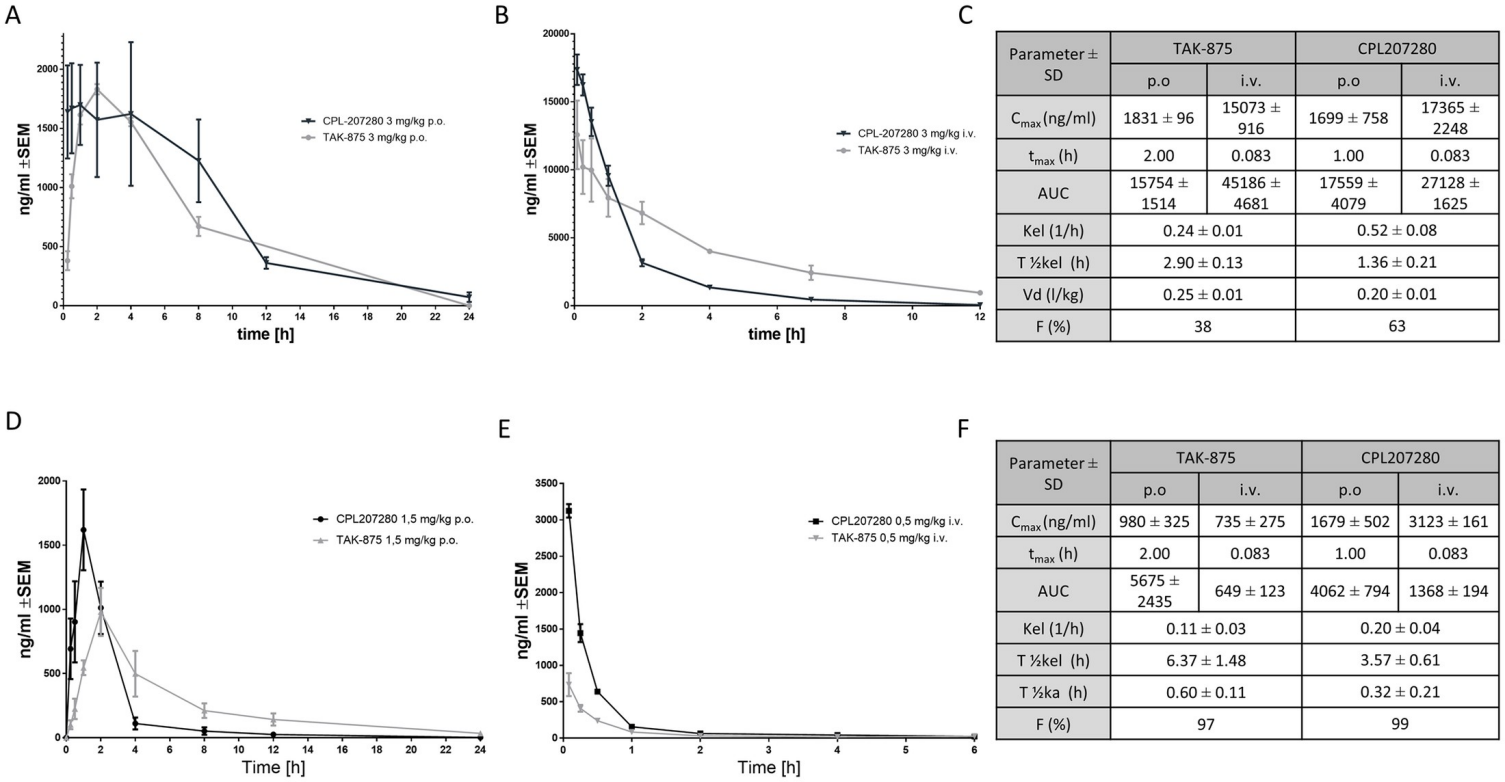
Pharmacokinetic profiles of CPL207280 and TAK-875. Plasma concentrations of CPL207280 and TAK-875 in rats (A, B, respectively) and monkeys (D, E, respectively). Pharmacokinetic parameters obtained in rats (C) and monkeys (F) after single oral (p.o.) and intravenous (i.v.) administration of doses. Note that in rats, both compounds show similar exposure—AUC and maximal concentration—C_max_, but differ significantly in terms of the bioavailability and clearance rate. Data are mean; n = 5 (rats); n = 3 (monkeys).

When doses of 40 to 600 mg/kg/day were administered, TAK-875 induced the accumulation of TBA in the circulation in rats and dogs and caused liver injury accompanied by elevation of ALT and bilirubin and no changes in other liver-injury markers [3, 25]. To study whether the same is possible for CPL207280 *in vivo*, we first tested whether counterpart doses ensured similar drug exposure in plasma. To address this question, a pharmacokinetic study comparing both molecules in animal models was performed. Rats were administered 3 mg/kg of compounds p.o. or the same dose i.v. CPL207280 demonstrated greater bioavailability (63%) than TAK-875 (38%) (Fig 5A and 5B).

**Fig 6:**
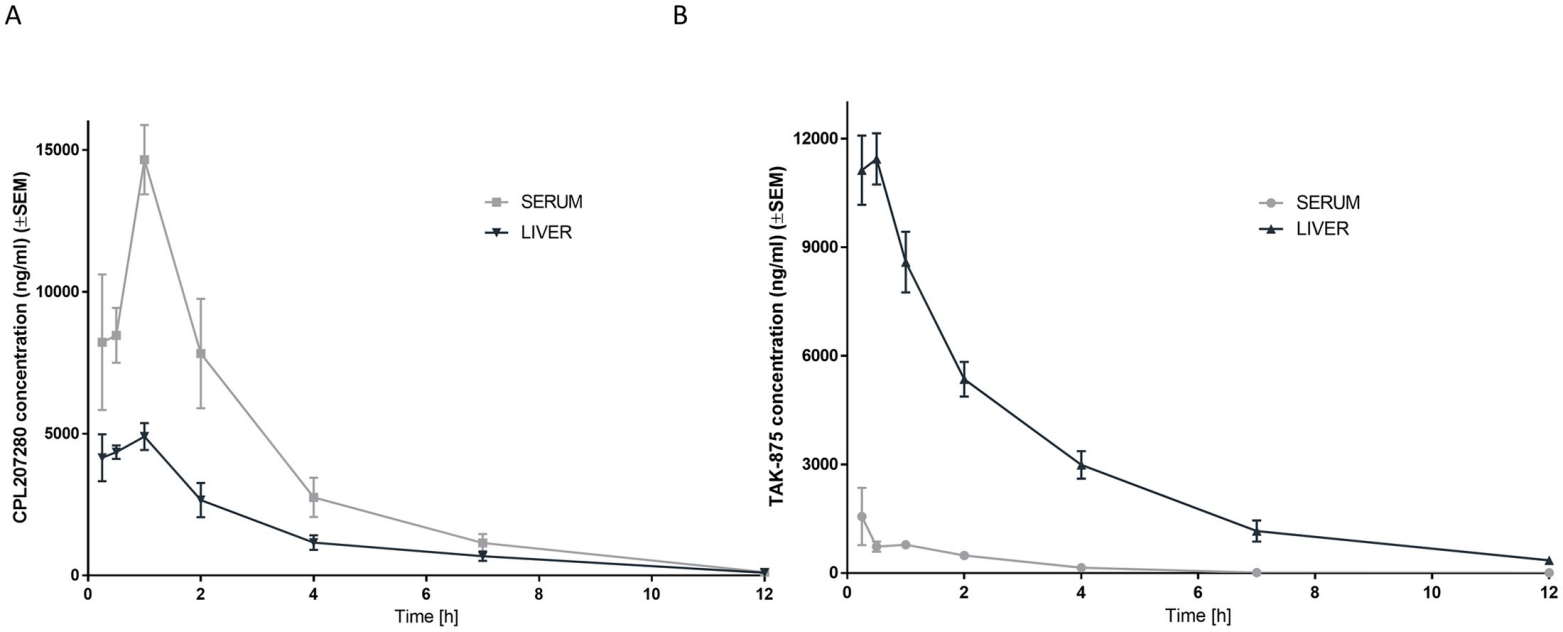
Pharmacokinetic profiles in mice. Concentrations of CPL207280 (A) and TAK-875 (B) in serum and liver. Data are represented as mean ±SEM (n = 3) concentrations of the compounds in mouse plasma and liver following a single oral administration. Note that CPL207280 shows greater concentrations in serum than in liver in contrast to TAK-875, which tends to mainly occur in the liver.

The latter also demonstrated a greater distribution volume (V_d_) of 20%, which is indicative of greater organ penetration. CPL207280 was quickly absorbed, reaching C_max_ in approximately 1 h and was quickly eliminated from circulation, which was reflected by 2x greater K_el_. TAK-875 reached C_max_ after 2 h (Fig 5C). Better bioavailability of CPL207280 but quick elimination resulted in only slightly greater exposure compared with that of TAK-875. Despite these differences in PK parameters, the overall exposure was comparable and generated adequate conditions for the comparison of toxic effects in rats. The smaller volume distribution for CPL207280 suggests that this compound does not accumulate in the liver, in contrast to TAK-875. To address this question, both compounds were administered p.o. to mice on one occasion. At the time points 15 min, 30 min, 1 h, 2 h, 4 h, 7 h, 12 h, and 24 h, the animals were euthanized and their liver specimens were collected, and the compound content was quantified. Indeed, TAK-875 tended to penetrate the liver more than serum as compared to CPL207280. For the latter serum was observed to be the central compartment (Fig 6A and 6B).

In monkeys, CPL207280 and TAK-875 administered on a single occasion showed similar bioavailability of approximately 99% (Fig 5D and 5E). Notably, CPL207280 demonstrated greater C_max_ when administered orally and intravenously. The half-life of CPL207280 was almost twice shorter than that of TAK-875 with i.v. administration. Eventually, the higher elimination rate led to a lesser exposure (AUC) to CPL207280 than to TAK-875 (Fig 5F).

### Repeat dose study in rats and monkeys—Pharmacokinetics

**Fig 7:**
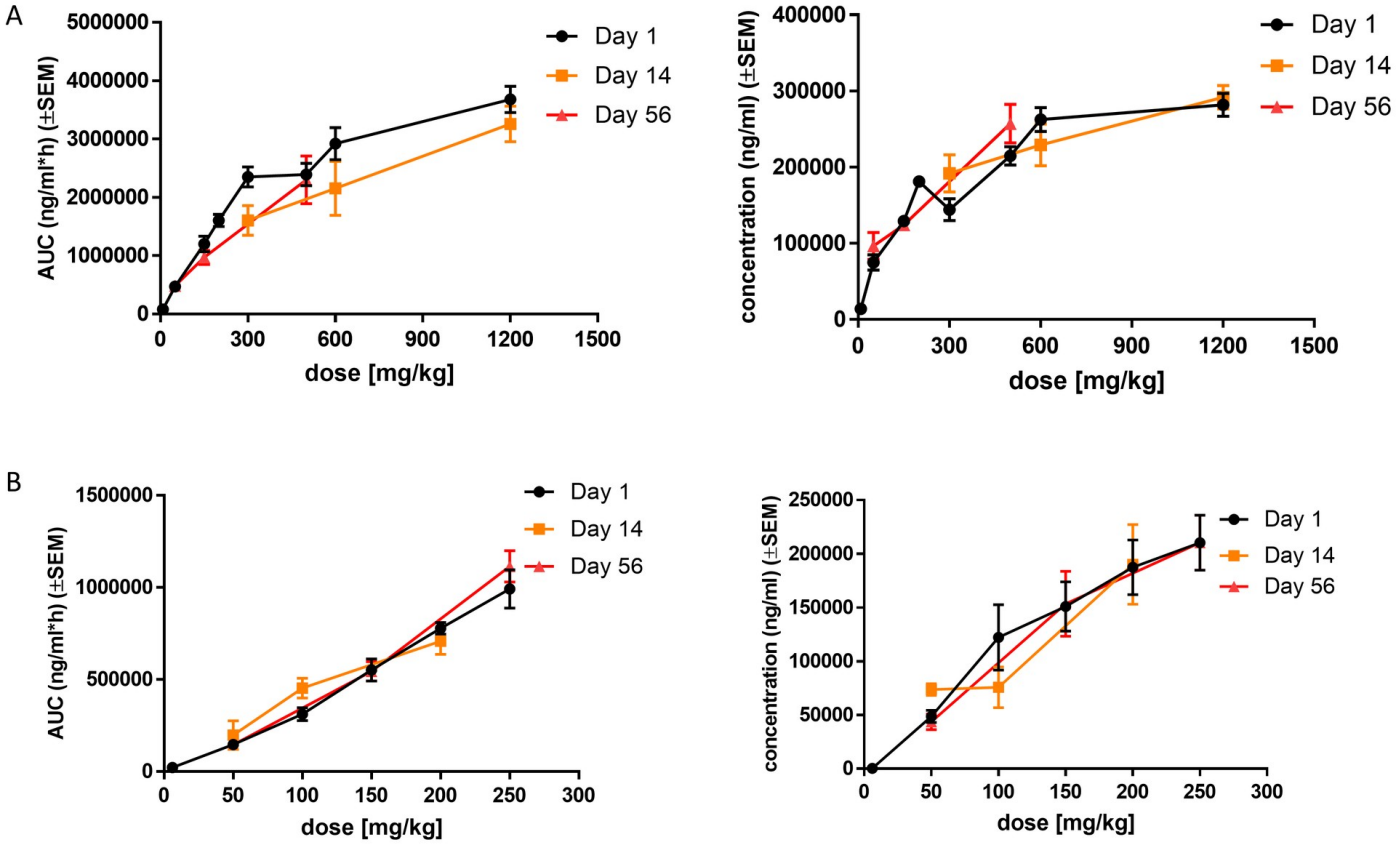
Pharmacokinetic profiles of CPL207280 in repeated-dose administration 14- and 56-day study in rats and monkeys. Wistar Han rats were orally given 300, 600 and 1200 mg/kg/day of the test compound for 14 days and 50, 150, 500 mg/kg/day (n = 6, 3 males + 3 females) for 56 days (A). Cynomolgus monkeys were orally given 50, 100, 200 mg/kg (n = 4, 2 males + 2 females) of the test compound and 50, 150, 250 mg/kg/day (n = 8, 4 males + 4 females) for 56 days (B). Plots represent dose–exposure and dose–C_max_ relationships. Data are mean ± SEM.

A previous pharmacokinetic study in monkeys and rats allowed the comparison of PK parameters of TAK-875 and CPL207280 administered on one occasion. Some stable compounds may accumulate in plasma when administered repeatedly. TAK-875 tended to accumulate in plasma of rats in a 14-day repeat-dose study [25]. For dose 200 mg/kg at day 1, TAK-875 exposure in plasma amounted to 1.21 mg*h/mL and increased to 3.74 at day 14. Similarly, for dose 600 mg/kg of TAK-875 at day 1, AUC was equal to 2.01 mg*h/mL and increased to 6.61 at day 14. This suggested accumulation of TAK-875 in the body, resulting in an increased pharmacodynamic response and potential toxicity. Following this observation, concentrations of CPL207280 in rat plasma at days 1, 14, and 56 were determined in the course of repeated administration of doses of 50, 150, 300, 500, 600, and 1200 mg/kg/day (Fig 7A). At day 1, there was a linear correlation between dose and exposure, as well as dose and C_max_ for doses up to 300 mg/kg. At higher doses, the PK values were lower than expected. At days 14 and 56, the exposure dropped insignificantly compared with that on day 1. This tendency was not observed for the C_max_. Next, the exposure and C_max_ values for doses 300 mg/kg and 600 mg/kg on days 1 and 14 with those for TAK-875 were compared (Table 4).

**Table 4:** The main pharmacokinetic parameters of CPL207280 compared with those of TAK-875 in rats in the repeat dose 14-day study (n = 6, 3 males and +3 females).

PK RAT	TAK-875	CPL207280	TAK-875	CPL207280	TAK-875	CPL207280	TAK-875	CPL207280
Day	1		1		14		14	
C_max_ (Mean) [ng/ml]	296000*	226910	465000*	256855	271000*	171795	383000*	194088
AUC24h (Mean) [ng x hr/mL]	1210000*	2350180	2010000*	2923280	3740000*	1605030	6610000*	2156620
Dose mg/kg/day	200*	300	600*	600	200*	300	600*	600

Data for TAK-875 (marked with “*”) were adopted from a previous study [25].

At day 1, CPL207280 demonstrated greater exposure by 50% than TAK-875 at a dose of 600 mg/kg. Further, the exposure was doubled at a dose of 300 mg/kg of CLP207280 than a dose of 200 mg/kg of TAK-875. Notably, increased exposure of TAK-875 was observed after 14 days, which exceeded the CPL207280 exposure by 200% for 600 mg/kg. The C_max_ remained unchanged. In monkeys, there was a clear dose-exposure and dose-C_max_ linear correlation. These dependences did not change after 14 and 56 days of treatment (Fig 7B). Because TAK-875 had different pharmacokinetics in monkeys when administered on a single occasion than CPL207280, the repeat dose pharmacokinetics for it were not carried out in this species.

### Repeat-dose study in rats—Safety

**Fig 8:**
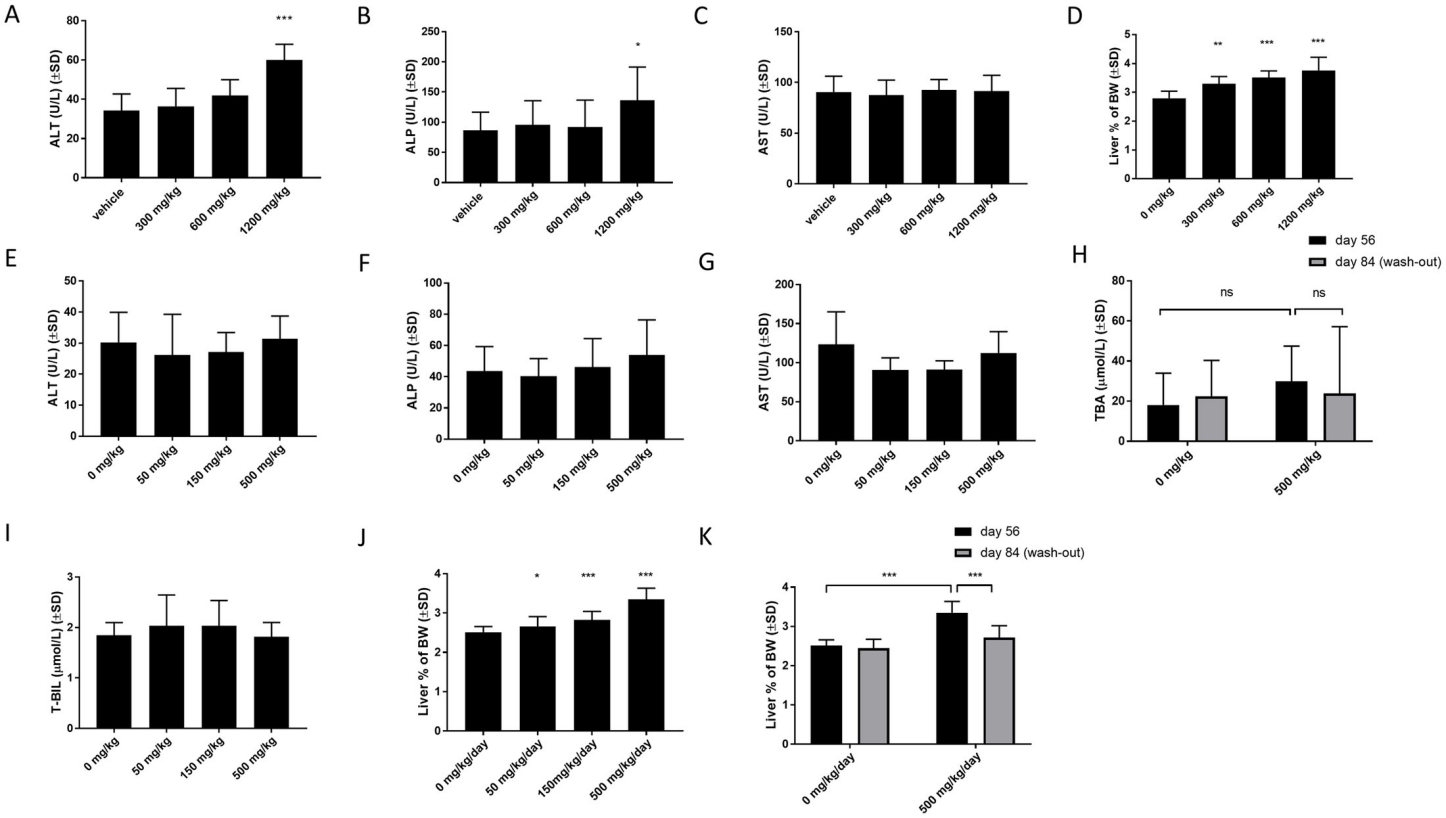
The effect of CPL207280 on liver toxicity markers in rats treated chronically. Serum levels of the biochemical markers (A-C) and liver mass (D) after 14 days of treatment. Data are mean ± SD, n = 10 (5 males + 5 females). Serum levels of the biochemical markers (E-I) and liver mass (J, K) in rats treated for 56 days. Statistical analysis was performed using One-way ANOVA, followed by Dunnett’s post-hoc test. Levels of TBA and changes in liver mass after wash-out were analyzed using Two-Way ANOVA followed by Sidak’s post-hoc test; ***, p< 0.001; **, p< 0.01; *, p< 0.05; n = 20 (10 males + 10 females). T-BIL—total bilirubin; TBA—total bile acids.

The preclinical safety of TAK-875 was studied in rats and dogs in a 14-day repeated-dose study because of the similar qualitative and quantitative metabolite profiles of these species and humans [25, 31, 36]. Treatment with TAK-875 dose-dependently induced elevation of ALT, bilirubin, and total bile acids after 7 days. Further, an effect of the same magnitude was observed at day 14. Given that CPL207280 demonstrated similar exposure to TAK-875 in rats after single administration, in the current study, rats were treated with the same dose range (0–600) and one dose of 1200 mg/kg doubling the highest dose studied in rats for TAK-875. After 14 days, liver injury markers ALT, ALP, AST in fasted blood were measured. After this time, the rats showed treatment-related adverse effects in the 1200 mg/kg group, corresponding to C_max_ = 250 μg/mL. Further, the death of one subject occurred on day 7. Necropsy showed decreased lymphoid cellularity in the splenic white pulp and diffused epithelial hyperplasia of the non-glandular gastric region, possibly related to death. The significant increases in ALT and ALP from 34.2±8.5 to 60.0±8.0 and from 86.3±30.2 to 136.1±55.3, respectively, were only observed in 1200 mg/kg group (Fig 8A–8C). Surprisingly, in all groups, CPL207280 evoked dose-dependent enlargement of the liver, expressed as a percentage of body weight (Fig 8D).

However, the tested liver injury markers did not change in CPL207280 groups treated with doses, in which they were significantly elevated due to TAK-875. In addition, histopathological examination revealed no microscopic changes in the liver tissue, except for a slight increase in its actual mass, suggesting no liver injury. The toxic effect of TAK-875 was described as dose and duration-dependent, that is, in dogs higher doses (150 mg/kg) caused liver injury after a shorter time (13-weeks), while liver injury was observed for lower doses (80 mg/kg) after a longer exposure of 39 weeks (3 times longer) [36]. To further analyze whether toxicity might occur after a longer repeat-dose regimen, the time of exposure was increased four times, up to 56 days (Fig 8E–8I). After this time, none of the previously monitored markers nor TBA and T-BIL were altered for the tested doses of 50, 150, and 500 mg/kg. Again, a slight increase in the liver mass was observed, and the histopathological analysis revealed mild to moderate hypertrophy of centrilobular hepatocytes at doses ≥ 150 mg/kg/day, correlating with an increase in liver weight (Fig 8J). This phenomenon was reversible after 28 days, suggesting an adaptive reaction of hepatocytes to the workload imposed by CPL207280 metabolism (Fig 8K).

### Repeat-dose study in monkeys—Safety

**Fig 9:**
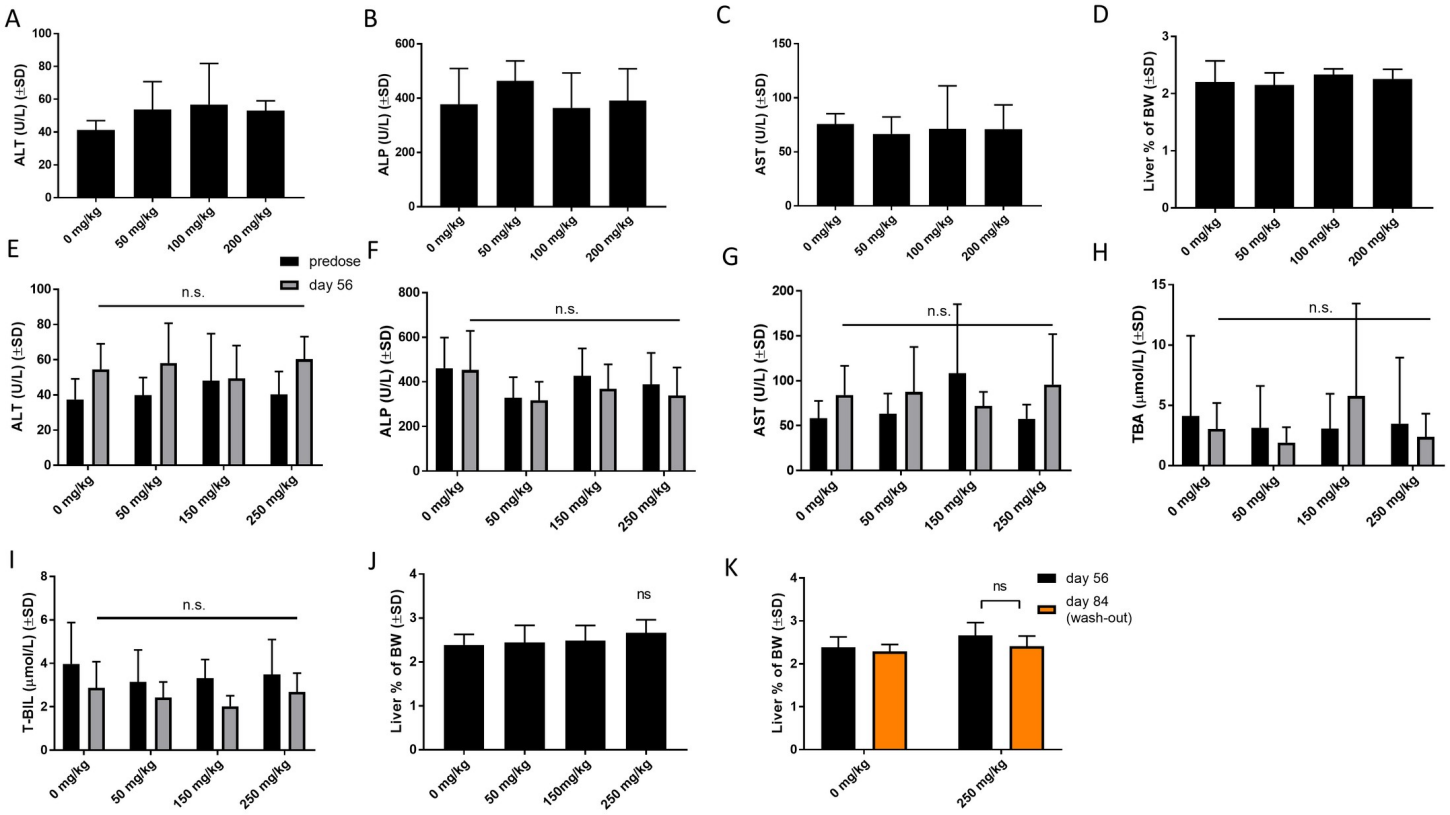
The effect of CPL207280 on liver toxicity markers in monkeys treated chronically. Serum levels of the biochemical markers (A-C) and liver mass (D) after 14 days of treatment. Data are mean ± SD, n = 4 (2 males + 2 females). Serum levels of the biochemical markers (E-I) and liver mass (J, K). Data are mean ± SD. Statistical analysis was performed using One-way ANOVA followed by Dunnett’s post-hoc test. Levels of TBA and changes in liver mass after wash-out were analyzed using Two-Way ANOVA followed by Sidak’s post-hoc test; n = 12 (6 males + 6 females) for doses 0 and 250 mg/kg; n = 8 (4 males + 4 females) for doses 50 and 150 mg/kg. T-BIL—total bilirubin; TBA—total bile acids.

As monkeys were the primary species whose hepatocytes showed a spectrum of CPL207280 metabolites similar to that observed for human hepatocytes, we decided to perform a repeat-dose study in a cynomolgus monkey. The doses were chosen based on the body surface area and amounted to 50, 100, and 200 mg/kg in the 14-day study and to 20, 150, and 250 mg/kg in the 56-day study [37]. No alteration of liver safety parameters was observed in monkeys within the tested dose range, that is, 50–250 mg/kg/day, irrespective of the treatment duration (Fig 9). Interestingly, neither hypertrophy nor increased liver mass was observed after the 14 and 56-day treatment.

### Repeat-dose study in diabetic rats and monkeys

**Fig 10:**
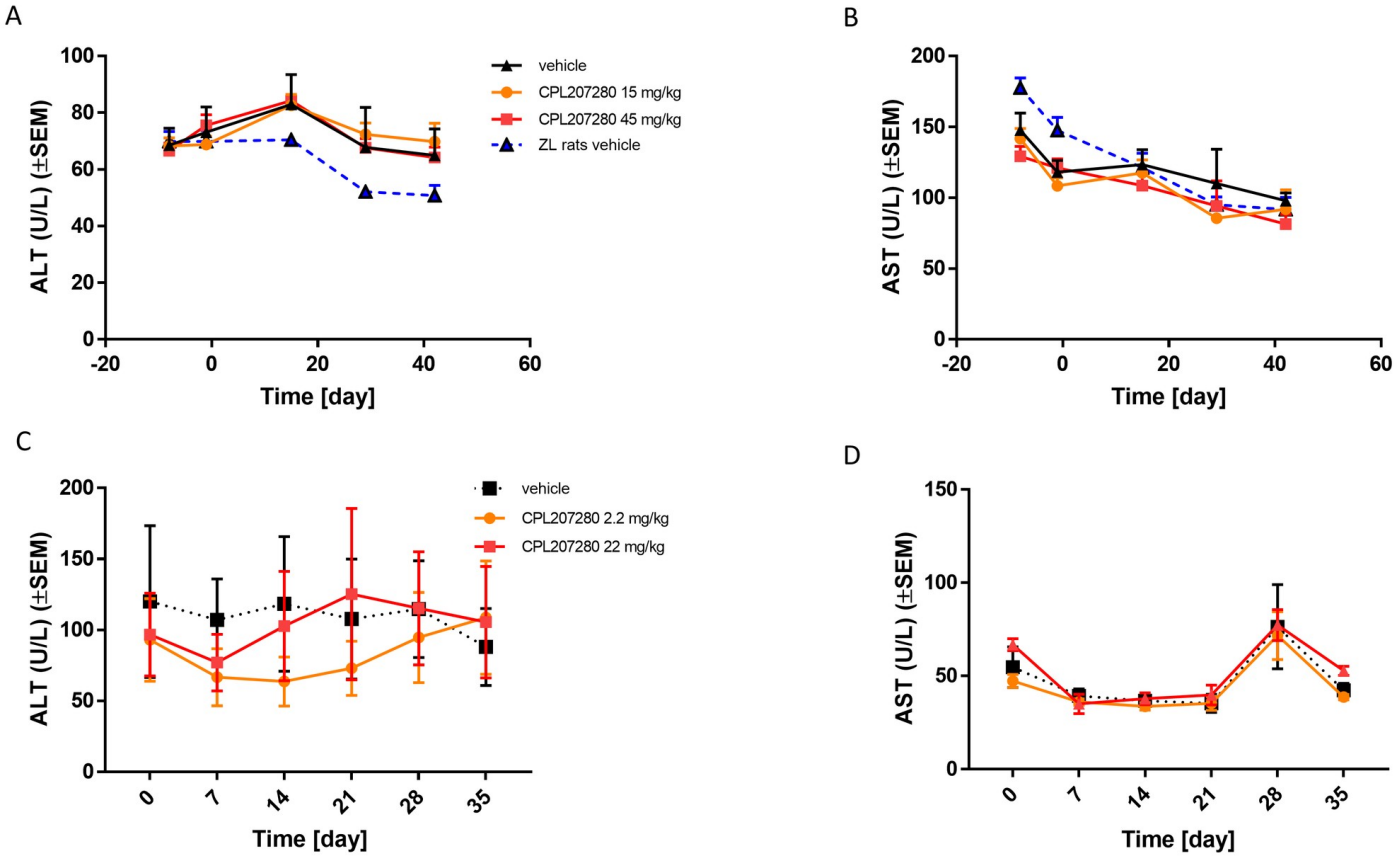
The effect of CPL207280 on liver toxicity markers in diabetic rats and monkeys. ZDF rats were treated with indicated doses for 42 days and serum was collected for ALT and AST analysis at different time points of the study (A, B). Diabetic cynomolgus monkeys were treated with indicated doses for 28 days followed by a 7-day wash-out (B). Serum was collected at different time points of the study for analysis of ALT and AST; Data are mean ± SEM; n = 8 for rats, n = 3 for monkeys.

T2D impairs liver metabolism. Hence, drugs that do not cause DILI in metabolically healthy subjects may still pose a risk of DILI in T2D individuals [38, 39]. To address this eventuality with regards to CPL207280, the safety of the compound was studied in Zucker diabetic fatty rats (ZDF) and diabetic cynomolgus monkeys challenged by chronic administration. ZDF rats constitute a genetically modified T2D model, in which leptin receptor deficiency and genetic defects of β-cells converge to induce severe T2D accompanied by its common comorbidities, such as fatty liver and steatosis. In contrast, only a fraction of cynomolgus monkeys develop insulin resistance and β-cell deficiency spontaneously when fed a high-fat diet; hence, their T2D is believed to have a similar etiology to that of humans [40, 41]. ZDF rats were fed a high-fat diet to induce diabetes. When the rats were 9 weeks old, treatment with CPL207280 commenced at doses of 15 and 45 mg/kg/day. ALT and AST levels were monitored at the start of the study after 15, 29, and 42 days. The markers changed over the course of the study for both diabetic ZDF and their lean healthy littermates, Zucker lean (ZL). Treatment with CPL207280 at any dose failed to alter the ALT and AST levels compared to the control vehicle-treated ZDF group (Fig 10A and 10B). The choice of the highest dose for the treatment of cynomolgus monkeys was based on allometric scaling and amounted to ½ of the highest dose in ZDF rats (22 mg/kg). The lowest dose was 2.2 mg/kg. Monkeys were treated for 28 days, after which the treatment was withdrawn, and monkeys were followed-up for an additional 7 days. Similar to ZDF rats, ALT and AST levels changed over time in both the treatment group and the vehicle group. Notably, none of the doses altered liver safety markers as compared to that of the vehicle group over 28 treatment days, suggesting intact liver metabolism, and its vitality (Fig 10C and 10D).

## Discussion

In this study, the liver safety of CPL207280, a novel GPR40 agonist developed for T2D treatment, was evaluated with a focus on addressing all toxicity-related concerns accounting for overall hepatotoxicity identified for the compound predecessor TAK-875 (Table 5). Interestingly, in this early drug-development stage, CPL207280 showed great liver-safety profiles in rats, monkeys, and human cells. Additionally, the study suggested that it is feasible to create a GPR40 agonist, which is safe for the liver and may circumvent mechanisms of toxicity known for the class represented by TAK-875.

**Table 5:** Summary of the comparative literature and features of CPL207280 and TAK-875.

	CPL207280	TAK-875
Toxicity for human hepatocytes	<	
Inhibition of bile acids transporters in liver	<	
Mitochondria Injury	<	
Acyl glucuronide presence in human hepatocytes	No	Yes
Bioavailability in rats	>	
Penetration into the liver	<	
Exposure in rat plasma for the same dose	≥	
Accumulation in rat plasma after repeat dose *	No	Yes
Propensity to elevate bile acids and bilirubin in plasma *	No	Yes
Propensity to elevate liver enzyme damage markers *	<	

*—[25].

In the current study, TAK-875 was used as a reference for CPL207280 to evaluate its safety properties: effect on viability of hepatocytes, inhibition of efflux bile acid transporters, production of reactive glucuronide metabolites, retention/deposition in the liver, and effect on liver safety markers of model animals. Recently, the liver safety of two GPR40 agonists—AMG-873 and TUG-770—was studied in mice and compared with TAK-875 [42]. It was found that only TAK-875 had a propensity for DILI and the other two were seemingly safe for the liver. However, the authors admitted that this study had limitations, as the actual drug exposure was unknown and might have been unequal despite using the same doses of all compounds. Similarly, in the other studies of ZYDG2 or HD-6277 researchers performed comparison of liver toxicity (including inhibition of bile transporters and toxicology study in rats and dogs, respectively) with that of TAK-875. However, the *in vivo* studies missed an equal drug-exposures to enable fair toxicity comparison [43, 44]. Drug-exposure divergences result from different absorption, distribution, metabolism, and excretion (ADME) of specific agonists in the studied animal species. In the current study, we ensured CPL207280 exposures similar to that of TAK-875 in previous study. Furthermore, the metabolic profile of CPL207280 justified choosing rat as the first model for toxicology, which was also the case for TAK-875. These aggregate properties enabled fair *in vivo* liver toxicity comparison [25]. However, the second species chosen for toxicology study of TAK-875 was a dog. In contrast, based on metabolite and compound clearance properties, monkey appeared as the appropriate second species for studying effects of CPL207280. This discrepancy in choice of the second model limited the *in vivo* comparison of both molecules to studies in rats only. The comparison in rats, however, was indirect and performed retrospectively via literature [25].

**Fig 11:**
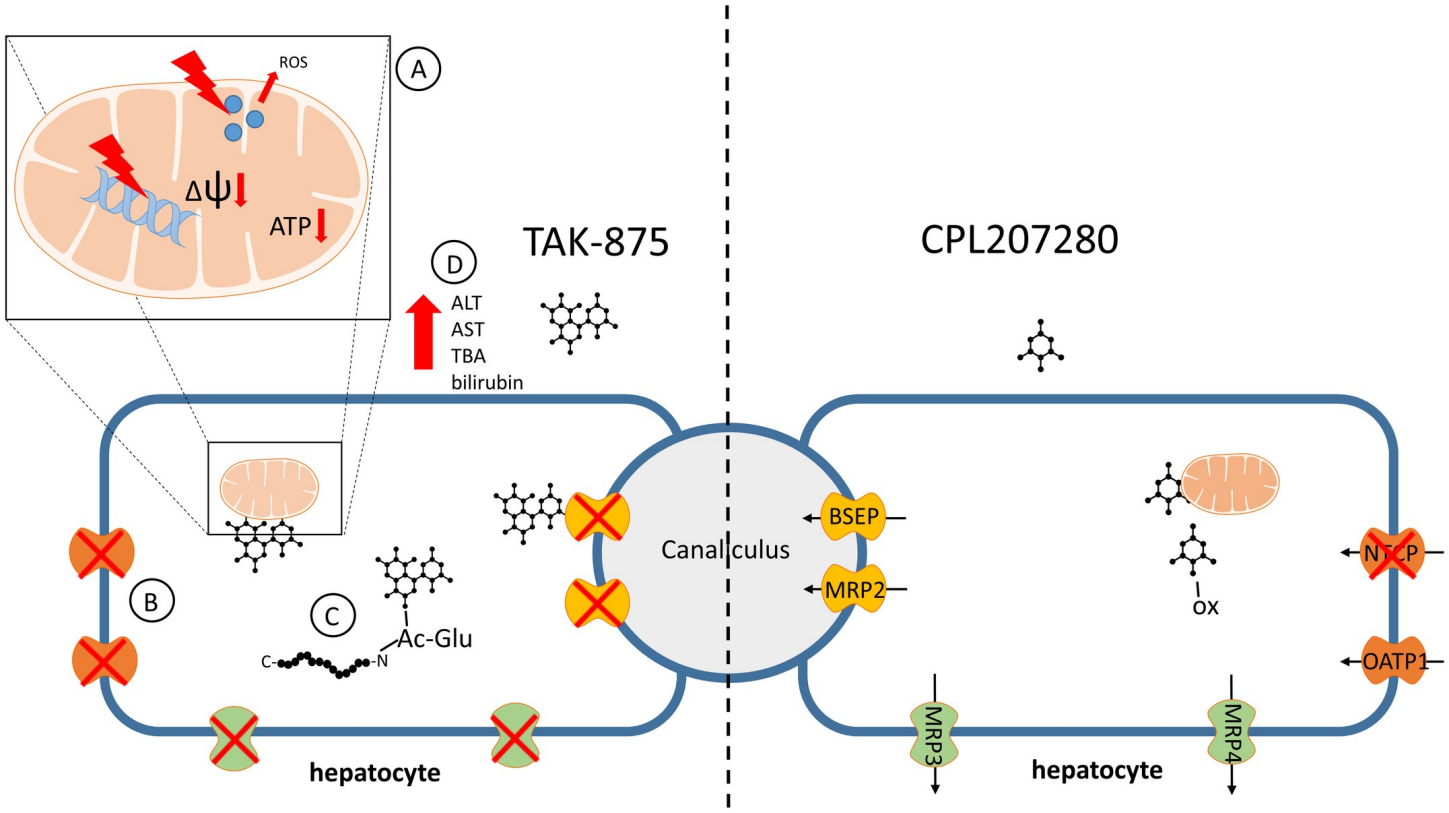
The scheme of potential processes that do not succumb to CPL207280 but are altered by TAK-875 leading to hepatotoxicity. In contrast to CPL207280, TAK-875 impairs hepatic mitochondria through DNA damage, ROS production, reducing inner membrane potential (Δψ), that precipitate the reduction of respiration (A); CPL207280 does not inhibit pivotal bile acid transporters, whereas TAK-875 abolishes their function thereby causes accumulation of bile acids in hepatocytes and their increase in circulation (B); CPL207280 is a small compound, which is quickly metabolized through an oxidation, yet bigger and more lipophilic TAK-875 forms reactive acyl-glucuronide, that has propensity to form covalent bonds with proteins whereby compromises enzymatic processes in the liver (C). All mentioned processes lead to the damage of the liver and increase ALT, AST, ALP, TBA and bilirubin in the circulation (D). TBA—total bile acids; The structures of depicted compounds are symbolic and not the actual, however, they reflect the difference in molecular weights.

The direct toxicity evaluation of CPL207280 and TAK-875 was enabled in *in vitro* experiments. Notably, starting with the study of the direct effect of test compounds on hepatocytes (HepG2 and human primary cells), we found that CPL207280 is 10 times better tolerated by hepatocytes than TAK-875. In the viability study, TAK-875 showed an IC_50_ of approximately 50 μM, which was in line with the values reported in initial studies detailing TAK-875 toxicity (Fig 1) [25]. This was also in agreement with a more recent study by others who reported IC_50_ values of 68 and 56 μM when the time of incubation was 24 and 48 h, respectively [45]. Authors underscored that the longer hepatocytes were incubated with the compound, the more sensitive they become to toxicity of the compound. Given that the time of incubation may influence sensitivity to compounds, we later studied CPL207280 in 3D cultures that permitted 14 day-long incubation periods (discussed further). Next, the inhibition of efflux bile acid transporters was studied in detail and compared for CPL207280 and TAK-875. Importantly, CPL207280 showed potency that was at least one order of magnitude less than TAK-875 for the inhibition of majority of transporters, which suggested 10 times lower toxicity that is in keeping with the effect observed in hepatocytes (Figs 1, 4 and 11B). The IC_50_ values for TAK-875 appeared very close to those reported in previous studies, except for NTCP [24, 25]. Inhibition of NTCP (51 μM) appeared weaker than the previously reported (4.6 or 2.0 μM). However, this may be a result of the poor confidence interval at 95%, measured for TAK-875 for this very transporter (Fig 4). NTCP is an influx transporter that directs bile acids from the portal vein into the hepatocytes. This process can be compensated by OATP1B1 and OATP1B3; therefore, it is unlikely that sole inhibition of NTCP at the level determined for TAK-875 might be of significance for the safety of CPL207280 [46]. The transporters that are more risky for liver safety include those belonging to efflux transporters, especially when compensatory action of the others is blocked. Of these transporters, BSEP and Mrp2 play a prominent role as they dispose of bile acids from hepatocytes [47, 48]. BSEP was inhibited by TAK-875 at IC_50_ of 47.3 μM and MRP2 at 48.1 μM, which falls in the range of IC_50_ assessed in the direct viability study in hepatocytes, implying that this mechanism may largely contribute to the observed toxicity *in vitro*. Importantly, CPL207280 showed no or very weak propensity to block either of the transporters. Taking into account the IC_50_ of TAK-875 in hepatocyte viability assays and the concentrations in human serum, it has been long debated whether TAK-875 induces hepatotoxicity in humans through inhibition of bile transport. This is because the TAK-875 did not reach the IC_50_ concentration in phase II or III [15, 16]. Moreover, the very high plasma protein binding (PPB) of TAK-875 (> 99.4%) further reduced its free plasma concentration levels, which is likely negligible for liver toxicity [31]. Nevertheless, it has been reported that TAK-875 accumulated in dog liver, thereby its local concentration might greatly exceed that of IC_50_, likely resulting in elevated bile acids in blood observed for both rats and dogs in the 14-day study [25, 36]. Importantly, such compound deposits were not observed in the current study focused on CPL207280 in either rat or monkey livers (not published). Although it is fair to assume that TAK-875 crystals were the cause of liver injury in humans, the exact mechanism of TAK-875-mediated DILI remains unclear, especially since the concentration of TBA has never been studied in the clinic. Recently, an elegant study combining mathematical modeling and *in vitro* experiments for evaluation of TAK-875 mechanisms of toxicity was carried out [49]. Simulations using PK data from human and *in vitro* toxicity data delivered similar outcomes to those observed in the clinic. Although ALT elevations were calculated, due to the limitations of the system, the authors were unsuccessful in obtaining TBA and T-BIL values. However, interestingly, the study showed that mitochondrial dysfunction and transporter inhibition must coexist to induce toxicity and that they have a synergistic effect. This finding underscores the importance of studying both mechanisms for toxicity inference. To address this scenario in the current work, respiration in hepatocytes was studied using the Seahorse XF platform. Notably, CPL207280 caused a slight respiration inhibition at 100 μM in contrast to TAK-875, which fully stopped oxidative respiration and tremendously reduced the spare capacity at the same concentration (Fig 2B). In a more detailed study in 3D spheroids, CPL207280 had no effect on mitochondria and DNA (Table 1, Fig 11A). In contrast, TAK-875 impaired mitochondrial function and caused DNA damage. TAK-875 also reduced glutathione content in the cells, and even though it was reported elsewhere of no account for TAK-875 toxicity, it might still underlie the observed elevated oxidative stress (Table 1) [45]. Regarding the deleterious effects of the parent molecule, it has been proposed that glucuronide (TAK-875-Glu), one of the main metabolites of TAK-875, might accumulate aside TAK-875 in the liver, thus causing additional injuries. Glucuronides are considered to be under special attention in the mechanisms underlying DILI [50]. They may form reactive species through the opening of the ring and sharing atoms for covalent bonds with proteins and DNA (Fig 11C). The chief metabolites of CPL207280 in human hepatocytes after 60-minute incubation appeared to be products of oxidation and accounted for over 73% of all metabolites. Importantly, glucuronide products appeared in negligible amounts, accounting for 6% of the total metabolites. Therefore, it was decided not to study CPL207280 glucuronide metabolite *in vitro* as it was unlikely that it might contribute to DILI in a significant manner. Furthermore, this metabolite was produced largely in monkey hepatocytes, so its toxicity was studied *in vivo* at concentrations greater than those expected in humans (Fig 3B). By contrast, TAK-875 and TAK-875-Glu were often investigated in parallel in a previous study. TAK-875-Glu turned out to be the main metabolite in dogs, rats and humans, and was studied alongside with its parent compound in numerous assays [31]. It is fair to note that TAK-875-Glu showed less toxicity in hepatocytes than its parent. It also demonstrated less potency in inhibition of bile acid transporters and was weaker at blocking phosphorylating oxidation and respiration [24, 25]. Recently, during the development of MK-8666 by Merck, a compound structurally similar to TAK-875, it has been shown that emerging glucuronide derivatives produce reactive acyl [51]. Indeed, glucuronide has an alarmingly short half-life, whereas only stable glucuronides with a half-life above 7 h are perceived as non-reactive, and thus non-toxic [52]. Furthermore, protein adducts covalently bound to MK-8666 metabolites have been found in hepatocytes. This scenario was considered a likely cause of the failure of MK-8666 in the clinical trial conducted on diabetic subjects, driving one patient into a state described by Hy’s law (ALT > 5x ULN and T-BIL > 2x ULN) [51].

In a previous study, it was observed that TAK-875 accumulated in rat plasma in a 14-day repeated dose study (Table 4). In the current study, CPL207280 did not accumulate in the plasma or liver. In contrast, TAK-875 tended to be retained in mouse livers in a single-dose study (Fig 6). Additionally, in rats, this compound showed a greater distribution volume (V_d_) than CPL207280 (Fig 5C). This subject was studied extensively in the previous work by calculating V_d_ in humans and rats, where even greater distribution volume in humans was observed [31]. It is fair to assume that this property may further propel mechanisms of toxicity by generating high concentrations locally. This may escalate in T2D subjects, whose livers are often exhausted by metabolic syndrome and thus they have less capacity to neutralize xenobiotic toxicity. Interestingly, in a 14-day study conducted in rats, no CPL207280 accumulation in plasma even at a dose above the maximum tolerated dose (MTD, 600 mg/kg) was observed (Fig 7). More importantly, no ALT, AST, and ALP elevation in a range of doses (up to 600 mg/kg) was observed, whereas TAK-875 showed significant elevation at doses of 200 mg/kg and greater [24, 25]. For TAK-875, this was associated with increases in T-BIL and TBA. In the second species (dogs), TAK-875 induced elevation of ALT and formed crystals in the livers of two of six animals treated with the highest dose (600 mg/kg). In contrast, in the second species (monkey) in a longer, 56-day study, CPL207280 did not alter liver injury indicators or T-BIL and TBA. This is of great importance, as these markers were recognized as tightly linked with TAK-875 liver toxicity, and their elevation might precede any alteration in ALT, AST, and ALP (Figs 8, 10 and 11D). In rats treated with CPL207280, minimal to mild centrilobular hepatocyte hypertrophy of the liver was noted, which fully subsided following the 28-day recovery period. Hepatocellular hypertrophy is frequently seen in the liver following exposure to agents that cause hepatic enzyme induction. In the present study, we considered an adaptive response due to detected induction of Cyp3a4 (data unpublished), rather than a pathological alteration. The limitation of the present study was that it did not cover the immune-toxicity aspect of CPL207280. In a recent study utilizing microarrays to study TAK-875 activated pathways in the liver, it was proposed that TAK-875 induces TLR-mediated immune response [42]. Moreover, it has been shown that TAK-875-Glu metabolite may evoke adaptive immune attack via Nrf2 response genes, which may contribute to toxicity [24]. Notably, for CPL207280, a negligible amount of the aforementioned metabolite was observed and hence CPL207280 is unlikely to cause an immune response. In addition, histopathology revealed no elevated immune cell infiltration in the livers of monkeys and rats (data not published). Nevertheless, the implication of the CPL207280 parent and its metabolites in inflammation should not be ruled out and warrants further investigation.

The exact mechanisms underlying TAK-875 and MK-8666 toxicity have not been fully elucidated. This phenomenon may rely on specific metabolic conditions in the liver of individuals with T2D. It must be emphasized that for both reference compounds, the toxicity in humans was observed in individuals burdened by metabolic syndrome and the toxicity rate observed was 2.7% within the first half-year of administration [20]. Subjects with certain genetic make-up may be more susceptible to DILI because of their liver metabolic limitations [38]. Therefore, a thorough study of the DILI is possible only in appropriate *in vivo* models representing the target syndrome, which is diabetes. To address this prerequisite condition, toxicity was studied in relevant diabetic rat and monkey models at effective doses [29]. The effects of TAK-875 in a diabetic rat model (ZDF) were studied elsewhere to demonstrate the efficacy and safety of the compound in β-cells after 6 weeks of treatment [53]. However, the authors did not publish liver-safety parameters. In the present study, the ZDF model was used to study the liver safety of CPL207280 in T2D. The ZDF rat is considered a high-end T2D model, which provides diabetic comorbidities that render it more similar to human disorders. Furthermore, a more robust monkey model of spontaneous T2D was used to support the findings from ZDF rats. Importantly, both models provided the target disease, CPL207280 metabolites, and ADME, as discussed previously. Chronic treatment with CPL207280 in the range of therapeutic doses failed to elevate ALT and AST in both species, suggesting that patients with T2D will be exposed to liver-safe doses of CPL207280.

## Conclusions

The main objective for the GPR40 agonist designers has been to devise compounds structurally different to FFAs and much more potent than FFAs [54, 55]. This was in order to avoid lipotoxicity, which may contribute to diabetes progression [56, 57]. In fact, synthetic agonists were reported not to be lipotoxic in the diabetic *milieu* (owing to beneficial activation of GPR40) [58, 59], but they eventually turned out toxic specifically to the liver. The accumulated body of evidence in this work suggests that CPL207280 is a peculiar GPR40 agonist, that significantly differs from other agonists, especially TAK-875 in terms of the structure and potency that determine liver safety in animal models. This feature provides a greater chance for safer use in the target population of patients with diabetes.

## Supporting information

S1 File (XLSX)
